# Medical and Obstetrical Cases

**Published:** 1846-05

**Authors:** Francis H. Gordon

**Affiliations:** Late President of Clinton College, Tennessee


March, 1846.

---

Art. III.—*Medical and Obstetrical Cases.* By Francis H. Gordon, M.D., late President of Clinton College, Tennessee.

*Retained Placenta.*—May 31st, 1844, I was called in haste to see Mrs. L., æt. 30 years, who had aborted twenty-four hours previously, being about three months and a half advanced. Two very old ladies, long practiced in midwifery, informed me that the difficulty was some unnatural growth in the womb. Upon examination, I pronounced it to be the placenta attached to the walls of the uterus; but the old women could not be convinced till I pinched off a portion and exhibited it to them. There was considerable peritoneal tenderness, the womb was not inclined to paroxysmal action, but quite sore, with its cervix dilated so as to admit the fingers with difficulty. Pulse feeble, and 110 per minute.—Gave stimulants to no advantage. Ergot, ʒss was administered every half hour, till three does were taken, without inducing uterine action. Tried artificial irritation, and external friction and pressure; but all was of no avail. Conceiving it impossible to extract the placenta by any plan that was safe to the woman, I determined to leave the matter to nature, unless future circumstances should demand interference. Directed one alvine evacuation to be procured daily with castor oil; a fourth of a grain of acetate of morphia to be given whenever she was restless; warm water to be freely thrown up the vagina every day; and a stream of warm water to be directed for three hours every day upon the abdomen, and to be followed by poultices of warm wheat bran, large enough to cover the whole surface.

No marked paroxysmal contraction ever took place, but the placenta gradually came away in a dissolved state, almost unconsciously to the patient; and her health gradually improved for about two months, when recovery was complete. She has since been delivered, at full term, of a healthy child.

*Tetanus from sudden arrest of the Catamenia.*—November 3d, 1843, I was called in the night to Miss V., æt. 16 years, of vigorous constitution. Two days before she had been caught in a shower of rain during the menstrual flow, which caused a sudden cessation of the secretion. On the 3d, at about 4 o'clock, P.M., she was suddenly seized with pain in the epigastrium, and tetanic spasms. I found her supine, every muscle rigid, and resting upon the occiput and heels, the trunk forming an arch between these extremities; her pulse nearly natural. The teeth continued so firmly clenched, that internal remedies were out of the question. I heated an iron poker, and the handle of a frying pan to a red heat, and using them alternately managed thus to keep a hot iron passing along the tract of the spine, within an inch of the skin, for about five minutes, when general relaxation suddenly ensued. The skin did not blister from the hot irons, though several spectators thought it must be literally roasted.

I treated the case with opium, sulph. ether, carb. ammonia, calomel and ipecac. The paroxysms, of about one hour's duration, returned at irregular intervals five or six times a day until the 12th, when the patient was left free from pain, soreness, or disease of any kind, being at once apparently in perfect health.

I should have remarked, that a blister was drawn with cantharides along the cervical and three or four dorsal vertebræ. One singular feature of this case was, that during the interval of relaxation the young woman displayed all the symptoms of nymphomania, and would not take a single dose of medicine unless it was given her by a young gentleman; evincing passionate fondness for any young man in five minutes who might chance to enter her room.

On the 13th I dismissed her cured, and directed warm teas, pediluvia, &c., to be used at several successive menstrual periods, so as to induce a regular return of the uterine secretion. The plan was successful as long as followed; but it was neglected at the fourth period, and I was summoned to see her on the 6th of March, 1844, in precisely the same condition as in November. Bled her 30 ounces; she vomited and was generally relaxed. Directed 6 grains of calomel and 2 grains of Dover's powder to be given every four hours until three doses were taken, to be followed by castor oil, and a blister to the nape of the neck. The spasms returned at irregular intervals till the 9th, when there was complete recovery. By being careful to promote menstruation at each regular period, the lady remained apparently healthy until the 22d of September, 1844, when I saw her laboring under an attack of autumnal fever. For two or three days she had spasms as in the other two attacks. A blister to the nape of the neck, and the usual treatment of fever, relieved her in a week, and her health has been excellent up to this time.

*Traumatic Neuralgia.*—Mrs. P. æt. 40 years, being far advanced in utero-gestation, was bled, June, 1843, in the foot, by a neighbor, and in the operation a branch of the external tarsal nerve was wounded.

August 17th. I was requested to visit her, and on doing so found her laboring under symptoms of hysteria; such as anxiety, flushed countenance, dyspnœa, and vertigo, with prominent eyes and vacant stare. The foot was swollen, and a little painful. Firm pressure along the course of the nerve gave relief, but a slight titillation caused intense pain. By this mode of procedure the wounded nerve could be traced to the sacrum. The patient also suffered from bronchocele, the tumor being so large as to obstruct the cerebral circulation. Friction along the vertebral column gave instant relief to the most distressing symptoms. Prescribed morphia and blue mass. Under the influence of these remedies she enjoyed partial comfort till the first of October, when she was delivered of a healthy child. Meantime iodine had been employed so as considerably to reduce the bronchocele. After delivery she suffered extremely, and in four weeks lost in weight more than seventy pounds. The pains were periodical, returning every evening with fever, and passing off towards morning with copious perspiration. The foot and entire limb were slightly swollen, and the whole length of the vertebral column was intolerant of heat or pressure. She slept but little, was anxious, restless, without appetite, and emaciating rapidly. Under these circumstances a caustic issue was established in the old cicatrix where the nerve had been wounded; the whole limb was bandaged, pustules from ointment of tartar emetic were kept up along the spine; half a grain of acetate of morphia was given whenever the patient was restless, or in much pain; two grains of sulphate of quinine were directed every two hours, from daylight till noon daily; and her bowels to be kept open with aloetic pills, alternated with blue mass and ipecacuan, *pro re nata.* Under this plan, the woman gradually improved; the pains almost ceased; her appetite returned; some increase of flesh was manifest; she slept well, and, in fine, considered herself rapidly convalescent.

Feb. 9th, 1844. She was attacked with typhoid fever, which brought back all the former symptoms with aggravated violence. One singular symtom now showed itself, surpassing anything I ever saw: the heart, as if hypertrophied, took on the most violent action, so as visibly to shake the patient all over, and to communicate a constant tremulous motion to her couch. It never ceased a moment while the patient lived. My excellent friend, Dr. Gowen, who attended the case with me in consultation, well described the phenomenon as "*a thundering of the heart.*" No treatment from this time afforded more than partial relief; and death occurred on the 26th, seventeen days after the supervention of fever.

*Lunar Caustic in Burns.*—During the autumn of 1840, while the writer was performing a set of experiments in chemistry before a class, a burn was accidentally received in the following manner. In getting out some phosphorus to light a spirit lamp, a little of it dropped into a box of mineral specimens under the table. Being unable to find it readily it was left in the box and forgotten until it got into a blaze. To save the minerals from injury, I attempted to run my left hand under them and the phosphorus and throw all out together; but instead of this, I ran the index and middle finger directly into the blazing phosphorus. The piece, about an inch long, stuck to my fingers, and before I could plunge them into the pneumatic trough, it completely roasted them from their extremities to the second joints. The pain exceeded in intensity any thing that ever I suffered.

But silver and its compounds being the subject of the lecture, there was fortunately a solution of the nitrate on the table, and as soon as reflection returned I plunged my fingers into the caustic liquid. Relief was prompt, and so great that in a few minutes the lecture was resumed. It was then about 3 o'clock, P.M.; there was slight pain in the fingers till 9, when I fell asleep and suffered no more from it afterwards. Hard crusts of sphacelus shielded the diseased parts till they were thrown off by reproduced flesh beneath. My fingers are now as perfect as ever in shape and motion, and no one could discover a cicatrix.

*Clinton College*, March, 1846.

